# Routine HIV Testing among Providers of HIV Care in the United States, 2009

**DOI:** 10.1371/journal.pone.0051231

**Published:** 2013-01-14

**Authors:** A. D. McNaghten, Eduardo E. Valverde, Janet M. Blair, Christopher H. Johnson, Mark S. Freedman, Patrick S. Sullivan

**Affiliations:** 1 Centers for Disease Control and Prevention, Atlanta, Georgia, United States of America; 2 Rollins School of Public Health, Emory University, Atlanta, Georgia, United States of America; Vanderbilt University, United States of America

## Abstract

In 2006, CDC recommended HIV screening as part of routine medical care for all persons aged 13–64 years. We examined adherence to the recommendations among a sample of HIV care providers in the US to determine if known providers of HIV care are offering routine HIV testing in outpatient settings.

Data were from the CDC's Medical Monitoring Project Provider Survey, administered to physicians, nurse practitioners and physician assistants from June-September 2009. We assessed bivariate associations between testing behaviors and provider and practice characteristics and used multivariate regression to determine factors associated with offering HIV screening to all patients aged 13–64 years.

Sixty percent of providers reported offering HIV screening to all patients 13 to 64 years of age. Being a nurse practitioner (aOR = 5.6, 95% CI = 2.6–11.9) compared to physician, age<39 (aOR = 1.9, 95% CI = 1.0–3.5) or 39–49 (aOR = 2.1, 95% CI = 1.4–3.3) compared with ≥50 years, and black race (aOR = 2.6, 95% CI = 1.2–6.0) compared with white race was associated with offering testing to all patients. Providers with low (aOR = 0.2, 95% CI = 0.1–0.3) or medium (aOR = 0.4, 95% CI = 0.2–0.6) HIV-infected patient loads were less likely to offer HIV testing to all patients compared with providers with high patient loads.

Many providers of HIV care are still conducting risk-based rather than routine testing. We found that provider profession, age, race, and HIV-infected patient load were associated with offering HIV testing. Health care providers should use patient encounters as an opportunity to offer routine HIV testing to patients as outlined in CDC's revised recommendations for HIV testing in health care settings.

## Introduction

The public health importance of identifying HIV-infected persons and linking them to care and treatment is three-fold: 1) providing care and antiretroviral therapy (ART) can improve the health outcomes of HIV-positive persons [1], [2]; 2) initiating ART may decrease transmission of HIV through the suppression of viral load [3]; and 3) awareness of one's positive HIV status often results in reductions in high-risk behavior that may transmit HIV [4], [5]. However, HIV is still diagnosed late in the course of disease for many people in the US: in 2008, 32% of persons with an HIV diagnosis received a diagnosis of AIDS within 12 months of their initial HIV diagnosis [6]. Implementation of routine HIV testing by health care providers presents an opportunity for diagnosis of HIV early in the course of disease.

Recommendations regarding who should be tested for HIV have evolved since HIV testing first became available in 1985. Early testing guidelines recommended testing for persons engaging in high-risk behaviors and those considered likely to be infected [7]. Later recommendations expanded to clinical settings including hospitals and emergency departments, particularly in higher prevalence areas [8], and pregnant women [9], [10]. Modifications to recommendations decreased barriers to the testing process, making the consent process easier and increasing access to testing in a variety of clinical settings. In 2001, CDC issued recommendations for routine HIV testing in clinical settings and of pregnant women [11] resulting in increased testing rates and identification of positives [12]–[14] and decreased effectiveness of targeted testing based on risk factors [15], [16]. Changes over time in populations affected by HIV provided strong rationale for the implementation of routine HIV testing in all health care settings. In 2006, CDC recommended HIV screening as part of routine medical care for all persons aged 13–64 years to decrease the number of people with undiagnosed HIV, diagnose HIV-infected persons at earlier stages of infection, link newly infected persons into care at earlier stages of infection, and prevent new infections [17].

The revised recommendations are for routine, opt-out screening for HIV for persons aged 13 to 64 in health care settings, including emergency departments [17]. As part of patients' general informed consent, similar to other diagnostic and screening or routine prenatal tests, oral or written information should be provided to notify patients that they will be tested unless they decline. Prevention counseling is no longer a part of routine HIV testing, but still is recommended for persons known to engage in behaviors that place them at risk for acquiring HIV. The guidelines recommend annual testing of persons considered to be at high risk for acquiring HIV, and state that diagnostic testing should be performed for patients with HIV-related signs or symptoms [17].

Although these guidelines have been in place since September 2006, minimal data have been presented on the adoption of routine testing by health care providers, particularly in outpatient settings where routine health care is delivered. We examined self-reported adherence to the CDC recommendations among a sample of HIV care providers in the US to determine if known providers of HIV care are offering routine HIV testing in outpatient settings.

## Materials and Methods

### Data Source and Sampling

Data were collected as part of the CDC's Medical Monitoring Project (MMP) Provider Survey, a survey administered to a nationally representative sample of HIV care providers who were selected to participate in MMP. MMP is an ongoing, supplemental surveillance system conducted in 20 states and six cities/counties that collects clinical and behavioral data on HIV-infected persons ≥18 years receiving care in the U.S. The methods of MMP have been previously described [18]–[20]. Briefly, MMP involves a three-stage sampling design. In the first stage, 20 states, which included >80% of the AIDS prevalence in the US (as of 2002), were sampled using probability proportional to size (PPS) sampling. Health departments in these 20 states and in six cities/counties within those states that are funded and administered separately for HIV/AIDS surveillance activities were funded to participate in MMP, resulting in 26 project areas. In the second stage, health department staff developed a facility sampling frame for their individual project areas. Facilities were eligible to be included on the frame if they were known to provide outpatient HIV care (defined as prescribing antiretroviral therapy or ordering CD4 or HIV viral load tests) to patients ≥18 years of age and managed their HIV-infected patients' medical care rather than referring them to other providers. A sample of facilities was selected from this facility sampling frame using PPS sampling. In the third stage, a sample of patients who received care at the sampled facilities during a specified time period was selected using a method that resulted in an equal probability of selection at the patient level.

The Provider Survey was administered to providers from the sampled facilities. Providers' contact information from participating sampled facilities was obtained by health department staff and each provider was assigned a unique MMP Provider Survey identification number. Based on available funding, a sub-sample of 2,000 providers was randomly selected from an estimated 2,550 individual providers participating in the MMP 2007 data collection cycle.

Health care providers eligible for the survey included physicians, nurse practitioners and physician assistants. Interns, residents and fellows in training programs were not included. Of the 2,000 health care providers sampled, 1,718 were eligible.

### Survey

The MMP Provider Survey was conducted from June through September 2009. Using the identification numbers, personalized recruitment packets containing a recruitment letter describing the purpose of the survey, information on how to access the survey online, a paper copy of the survey with a postage-paid return envelope, and a $15 gift card were sent to the sampled providers. The Total Design Method [21], [22] was used to increase response to the survey. Reminder postcards were sent to all sampled providers one week after the initial mailing, and a replacement survey, a copy of the original recruitment letter, and a letter for non-responders were sent to all providers who had not completed the survey at three and seven weeks following the initial mailing.

The survey included questions about provider demographics, length of time in practice, self-assessed knowledge about HIV, practice characteristics, and offering HIV testing to patients. Provider demographics included profession, board certification, age, gender, race, ethnicity, length of time providing care for patients with HIV/AIDS, whether they considered themselves to be a specialist in the treatment of HIV/AIDS, and how knowledgeable they considered themselves to be regarding HIV treatment. Practice characteristics included how often they refer their HIV patients to another provider with specialized knowledge of HIV care and treatment and the number of patients with and without HIV/AIDS to whom the provider gave care during an average month. For the analysis, the number of HIV-infected patients providers reported caring for per month was categorized as low (1–19 patients), medium (20–74 patients) or high (≥75 patients). Providers were asked to provide an estimate of the percentage of their patients living with HIV/AIDS by race, ethnicity, gender, and the percentage that injected drugs or were men who had sex with other men. To assess adherence to CDC's recommendations for routine testing, the following question was asked: “CDC recently recommended HIV screening in health care settings for all patients 13 to 64 years of age. Do you offer HIV screening to your patients?” The response options were: 1) Yes, to all my patients 13 to 64 years of age; 2) Yes, but mainly to patients who engage in high-risk behaviors; 3) No, but I plan to start offering HIV screening for all my patients 13 to 64 years of age; 4) No, I do not think HIV screening is necessary for all my patients 13 to 64 years of age; and 5) Not applicable, as I only see patients living with HIV/AIDS. For the purpose of this analysis, response options were dichotomized as 1) “Test All Patients”, which included only response 1 (“Yes, to all my patients 13 to 64 years of age”), and 2) “Do Not Test All Patients”, which included responses 2 through 4 above.

### Ethics Statement

CDC's National Center for HIV, Viral Hepatitis, STD and TB Prevention (NCHHSTP) has determined that MMP is a public health surveillance, non-research activity used for disease control program or policy purposes. Because NCHHSTP has determined MMP is not research, it is not subject to human subjects regulations including federal investigational review board (IRB). As an amendment to MMP, the MMP Provider Survey is covered under the same non-research determination. Participating project areas obtain IRB approval as required in each jurisdiction to conduct MMP.

### Analyses

Our analysis was restricted to providers whose practices included HIV negative patients, as those with only HIV-infected patients would not need to test. Chi-square analysis was used to assess bivariate associations between testing behaviors and provider and practice characteristics. Analysis of board certification was limited to bivariate analysis since eligibility for certification was dependent on provider type. Factors significantly associated with offering HIV screening to all patients aged 13–64 years (p<.10) were included in multivariate regression models using backwards stepwise regression, and adjusted odds ratios (aORs) and corresponding 95% confidence intervals (CIs) were computed. Due to low patient-level response rates, analysis weights were not derived for 2007 MMP data. Analyses were conducted using SAS version 9.1 (SAS Institute, Cary, NC).

## Results

Surveys were returned by 735 (42%) providers; 506 (69%) had HIV-uninfected patients in their patient population and were included in the analyses. Sixty percent reported offering HIV screening to all patients 13 to 64 years of age. Thirty-one percent reported offering screening mainly to those at high risk, and 9% reported not offering HIV screening to patients. Table **1** presents the characteristics of the providers by HIV testing behaviors. When asked to describe characteristics of their HIV-infected patient population, providers reported on average that 40% of patients were black, 22% Hispanic, 34% white, 30% women, 18% injection drug users (IDUs), and 59% were men who have sex with men (MSM) (data not shown).

**Table 1:** Characteristics of HIV care providers by testing behaviors – United States, Medical Monitoring Project Provider Survey, 2009.

Characteristic	Number (%)	Test All Patients N (%)		p-value
Total Providers	506	302	(60)	
Profession				
Physician	401 (79)	218	(54)	<0.001
Nurse Practitioner	68 (14)	58	(85)	
Physician Assistant	37 (7)	26	(72)	
Physician Infectious Disease Board Certified				
Yes	226 (59)	112	(50)	<0.01
No	160 (41)	100	(63)	
Age (years)				
<39	78 (15)	50	(64)	0.004
39–49	189 (38)	125	(67)	
≥50	234 (47)	121	(52)	
Gender				
Male	290 (58)	152	(52)	<0.001
Female	214 (42)	149	(70)	
Race/Ethnicity				
Black	43 (9)	33	(77)	0.006
Hispanic*	49 (10)	37	(75)	
White	359 (71)	201	(56)	
Other	51 (10)	29	(57)	
Time Caring for HIV+ Patients (years)				
≤5	65 (13)	38	(59)	0.03
6–10	105 (21)	74	(70)	
>10	328 (66)	183	(56)	
Specialist in HIV Treatment				
Yes	378 (78)	234	(62)	0.03
No	109 (22)	55	(50)	
Knowledge in HIV Treatment				
Extremely	193 (38)	127	(66)	0.04
Very	223 (44)	131	(59)	
Somewhat	83 (17)	40	(48)	
Not at all	6 (1)	3	(50)	
Number HIV+ Patients Provide Care to per Month				
1–19 (low)	116 (24)	48	(41)	<0.001
20–74 (medium)	206 (41)	113	(55)	
≥75 (high)	172 (35)	135	(78)	
Percent Black HIV+ Patients				
<25%	177 (37)	97	(55)	0.18
≥25%	305 (63)	186	(61)	
Percent Hispanic HIV+ Patients				
<25%	337 (69)	195	(58)	0.56
≥25%	145 (31)	88	(61)	
Percent White HIV+ Patients				
<25%	174 (37)	113	(65)	0.04
≥25%	308 (63)	170	(55)	
Percent Women HIV+ Patients				
<25%	233 (46)	136	(58)	0.62
≥25%	271 (54)	164	(60)	
Percent IDU HIV+ Patients				
<25%	364 (72)	217	(60)	0.95
≥25%	140 (28)	83	(59)	
Percent MSM HIV+ Patients				
<25%	67 (13)	41	(61)	0.79
≥25%	432 (87)	257	(59)	

* Hispanic persons may be of any race.

In bivariate analyses we found significant differences in reporting offering HIV tests to all patients 13 to 64 years of age by profession, infectious disease board certification, age, gender, race/ethnicity, time providing care for HIV-infected patients, whether providers considered themselves specialists in the treatment of HIV/AIDS, providers' self-reported knowledge of HIV treatment, number of HIV-infected patients providers care for, and percent of providers' HIV-infected patients that were white. With the exception of white race, differences in the characteristics of providers' HIV-infected patients, including race, ethnicity, gender and risk behaviors, were not significant (Table **1**).

In multivariate analysis, being a nurse practitioner (aOR = 5.6, 95% CI = 2.6–11.9) compared to physician, age <39 (aOR = 1.9, 95% CI = 1.0–3.5) or 39–49 (aOR = 2.1, 95% CI = 1.4–3.3) compared with age ≥50 years, and black race (aOR = 2.6, 95% CI = 1.2–6.0) compared with white race was associated with offering HIV testing to all patients. Providers having an HIV-infected patient load per month that was low (aOR = 0.2, 95% CI = 0.1–0.3) or medium (aOR = 0.4, 95% CI = 0.2–0.6) were less likely to offer HIV testing to all patients according to the recommendations compared with providers with high HIV-infected patient loads (Table **2**).

**Table 2:** Factors associated with HIV care providers offering HIV testing to all patients – United States, Medical Monitoring Project Provider Survey, 2009.

Characteristic	Adjusted Odds Ratio (95% Confidence Interval)	p-value
Profession		
Physician	Reference	
Nurse Practitioner	5.6 (2.6–11.9)	<0.001
Physician Assistant	1.7 (0.8–3.9)	0.23
Age (years)		
<39	1.9 (1.0–3.5)	0.03
39–49	2.1 (1.4–3.3)	<0.001
≥50	Reference	
Race/Ethnicity		
Black	2.6 (1.2–6.0)	0.02
Hispanic	2.0 (0.9–4.2)	0.09
White	Reference	
Other	1.0 (0.5–2.0)	0.94
Average Number HIV+ Patients per Month		
1–19 (Low)	0.2 (0.1–0.3)	<0.001
20–74 (Medium)	0.4 (0.2–0.6)	<0.001
75 or more (High)	Reference	

## Discussion

Sixty percent of HIV care providers who responded to the MMP Provider Survey reported offering routine HIV screening to all patients 13 to 64 years of age. Nurse practitioners, providers aged <50 years, black providers, and providers with high HIV-infected patient loads were more likely to offer HIV testing to all patients according to the recommendations. Although there is limited information regarding the characteristics of providers who conduct routine HIV testing, there is evidence that physicians who had previously diagnosed an HIV-infected person were more likely to offer HIV testing to their patients [23]. Our data extend this finding, by reporting that as the number of HIV-infected patients a provider cares for increases, the likelihood of providing routine testing increases.

No significant differences were found by characteristics of providers' HIV-infected patients. A higher percentage of providers (65% versus 55%) reported offering testing to all patients if their percent of white patients with HIV was less than 25% compared with ≥25%, but this was not significant in the multivariate analysis. However, others have found differences in routine offering of HIV testing by age, race and ethnicity of patients. Myers et al. found that blacks were more likely than whites to be offered testing, and Latinos and persons of other racial/ethnic groups (not Latino, white or black) were less likely to be offered testing [24]. The same study found that patients aged less than 55 years, and most notably men aged less than 18 years, were less likely to be offered testing.

Since the release of the CDC recommendations, implementation and acceptance of routine HIV screening programs has been successful in a variety of settings; most reports have been from hospitals or emergency departments. In the first 8 months following the October 2006 implementation of a hospital-wide routine rapid HIV testing program at Howard University Hospital, 57% of 9,810 patients who were offered testing agreed to test [25]. In a District of Columbia emergency department (ED) where trained HIV screeners offered rapid testing to 4,187 patients treated in the ED during a 3 month period, nearly 60% accepted testing [26]. Routine HIV screening was offered to 954 individuals in three South Carolina community health clinics starting in December 2006, with reported acceptance rates of 62%, 56% and 47%, respectively, in the first 8 months [27]. Other attempts at implementing routine testing have not been as successful. Routine testing was offered to 3,467 patients in a District of Columbia Veterans Affairs (VA) hospital from November 2007 through March 2009, but only 25% accepted [28]. Similarly, a Denver emergency department offered routine opt-out testing to 28,043 patients from April 2007 through April 2008, with only 24% accepting testing [29]. In other facilities and health care systems, routine HIV testing has yet to be implemented. A survey of veterans conducted from mid-October 2008 through mid-February 2009 found that HIV testing is not being routinely offered by VA providers. Of over 31,000 survey respondents, only 9% said they had been offered an HIV test in the past 12 months [30]. Further, a 2009 online survey of MSM in the US found that only about half of HIV-negative MSM reported being offered an HIV test by their routine health care provider in the past year [31].

Although we did not ask providers their reasons for not conducting routine HIV testing, several barriers to implementing the recommendations have been documented [32] and are likely similar to barriers experienced by our survey respondents. Barriers identified include: state and federal agency laws [33]–[35]; providers' concern about lack of prevention counseling [32], [33]; stigma and discrimination associated with HIV [33], [34]; and the perception that conducting risk-based testing is more cost effective than routine testing [36].

CDC acknowledged in the recommendations that state statutes and clinic policies might pose barriers to fully implementing the recommendations [17]. These barriers were found by Mahajan and colleagues when they examined whether implementing the CDC recommendations was compatible with individual state statutes during the two years following the release of the recommendations [37]. They reported that 16 states had statutes that were inconsistent with the key features of the recommendations: 1) opt-out testing; 2) informed consent; and 3) lack of HIV prevention counseling, meaning that implementing one or more of the provisions of the recommendations could not occur without amending existing laws. In the two years following the release of the recommendations, nine of the 16 states passed laws that were consistent [37]; in 2010, six states still had laws inconsistent with the recommendations [38]. Massachusetts, Michigan, Nebraska, New York (with the exception of rapid testing) and Pennsylvania still required specific written consent for HIV testing, and Michigan, Pennsylvania and West Virginia still required post-test counseling for a negative or positive result. In our survey provider sample sizes were inadequate to assess state as a predictor to determine the impact of state and clinic policies on implementing the recommendations.

Barriers to testing among physicians include pre-test counseling requirements, lack of knowledge of testing recommendations and requirements and lack of training in conducting HIV testing, lack of time, lack of acceptance by patients, burden of the consent process, language barriers, competing priorities and inadequate compensation [39]. Lack of training may be less of a barrier for younger providers, who have likely received more training regarding HIV testing, diagnosis and treatment than their older counterparts. This is supported by our finding that younger providers were more likely to offer testing. Lack of knowledge of the recommendations was identified by a survey of internal medicine residents in New York City conducted in early 2007 between five and nine months after the release of the recommendations. Only 32% of those surveyed were aware of the recommendations, and most were not offering routine testing; 36% of the residents used a routine testing approach, while 64% reported utilizing risk-based testing [40]. Sixty-eight percent said they would order more HIV tests if consent were oral rather than written and 46% had consent issues (written consent was required by New York State law at the time), 41% reported lack of time, and 20% cited language barriers. Factors associated with ordering 10 or fewer tests included lack of pre-test counseling training, conducting risk-based testing, and taking sexual history never or occasionally. In addition to the barriers above, lack of reimbursement for the test and lack of capacity to provide services or medications for patients with newly diagnosed HIV may also be reasons that testing is not offered.

If recommendations for HIV screening are routinely implemented, high percentages of patients offered testing would accept. Although lack of patient acceptance was noted by physicians [39], the literature shows that when more patients are offered HIV screening, more patients are tested. Six health centers that provide primary care and prevention services to underserved populations in North Carolina, South Carolina and Mississippi conducted almost 3 times the number of HIV tests after implementing routine testing compared with the previous year [24]. Of the 9% of VA patients mentioned above who said they had been offered an HIV test in the past 12 months, 91% accepted [30]. A survey to assess patients' acceptance of opt-out HIV testing in an urban emergency department asked 529 patients if they would accept opt-out HIV testing; 81% reported they would have accepted [41]. In the VA survey mentioned above, 73% of respondents reported they would be “very likely” to accept HIV testing, if recommended by their doctor [30], and focus groups at VA facilities found that both patients and providers agreed that routine testing would be beneficial to public health and to patients [42].

Although cost may be perceived to be a barrier to offering routine testing, several recent studies have found routine opt-out testing to be cost-effective [26], [43]–[45]. Holtgrave found that risk-based testing would result in more HIV diagnoses and prevent more HIV infections at a lower cost compared with routine opt-out testing, but in turn would increase the cost burden on the health care system to provide medical care to these newly identified persons [36].

Overall, HIV testing has increased in the US since the guidelines were introduced, but awareness of the guidelines, the importance of routine HIV testing, and current testing coverage still needs to be conveyed to providers of HIV care and other medical care providers in the US. The baseline for evaluating the effects of CDC's recommendations was developed from the National Health Interview Survey (NHIS). This survey determined that in 2006, an estimated 40.4% (71.5 million) of adults aged 18–64 years in the US reported ever receiving HIV testing [46]. Also using NHIS data, CDC reported that among persons aged 18–64 years in 2009, 45.0% (82.9 million) reported ever receiving an HIV test [47]. While this increase is encouraging, it still means that by 2009, 55% of persons aged 18–64 years had never received an HIV test. These numbers will need to increase at a faster rate to meet the National HIV/AIDS Strategy goal of increasing the percentage of persons with HIV who are aware of their status to 90% by 2015 [48]. In addition to the increased resources needed to implement routine HIV testing, the identification of new HIV cases will require linkages to HIV care and treatment resulting in in further demand on our nation's health care system. However, these additional resources required in the short-term will result in savings in costs and lives in the long-term.

There are several limitations to our study. Cost constraints prohibited selecting all 2,550 individual providers participating in the 2007 MMP data collection cycle. Our response rate of 42% was low; however, our sample included mostly physicians, who have lower survey response rates compared to non-physicians [49]. Results are based on providers' self-reported responses to survey questions. Given that these are providers of HIV care with the majority caring for patients >10 years, who consider themselves experts in and knowledgeable of HIV treatment, we expected high rates of screening to be offered to their HIV-negative patients. Providers' estimates of the number of HIV-infected patients they provide care to and the racial, ethnic and behavioral characteristics of their HIV-infected patient populations were likely self-determined and not derived using clinic records. We have previously experienced errors in estimates of patient loads during the construction of facility sampling frames in MMP [20]. Providers sampled and who responded to the survey may not be representative of HIV care providers in the US, and therefore, results may not be generalizable. We recommend that provider surveys that are conducted in the future as part of MMP include all sampled providers and that analysis weights are incorporated to adjust for selection probabilities and nonresponse.

## Conclusions

Although providers of HIV care likely have an increased awareness of the benefits of routine HIV screening and early identification of HIV-infected patients, many are still conducting risk-based rather than routine testing. Among HIV care providers surveyed, we found that provider profession, age, race, and HIV-infected patient load were associated with offering HIV testing. Based on our finding that 60% of HIV care providers reported offering routine HIV testing to all patients, we recommend that health care providers use patient encounters as an opportunity to offer routine HIV testing to patients as outlined in CDC's revised recommendations for HIV testing in health care settings to increase the number of patients tested. Organizations for HIV medical professionals are uniquely suited to increase awareness of the guidelines and encourage their members to routinely offer HIV testing to their patients. Assessing and reporting HIV testing practices at the state and local level could also provide an opportunity to increase awareness and monitor adherence to the guidelines, particularly among providers with few or no HIV-infected patients. The existing health care system will require additional resources to fully implement the recommendations to provide routine opt-out HIV testing. Resources will be needed not only to conduct additional tests, but to provide medical care to the increased number of persons with newly diagnosed HIV infection. However, the increased initial costs of routine HIV testing can be offset by the improved health outcomes associated with identifying HIV early in the disease course, providing timely entry into medical care, and the potential to prevent new infections through suppression of HIV viral load and patients' awareness of their HIV status.
